# The Effectiveness of Endovenous Thermal Ablation for the Knee Symptoms of the Osteoarthritis with Varicose Veins

**DOI:** 10.3400/avd.oa.21-00016

**Published:** 2021-06-25

**Authors:** Yuki Oga, Satoru Sugiyama, Susumu Matsubara, Yasuhiko Inaki, Masashi Matsunaga, Akira Shindo

**Affiliations:** 1 Department of Surgery, Hiroshima Teishin Hospital, Hiroshima, Hiroshima, Japan; 2 Radiology, Hiroshima Teishin Hospital, Hiroshima, Hiroshima, Japan; 3 Orthopedics, Hiroshima Teishin Hospital, Hiroshima, Hiroshima, Japan

**Keywords:** varicose vein, knee osteoarthritis, knee joint pain, endovascular ablation, subjective symptom

## Abstract

Patients with varicose veins of the lower extremities with osteoarthritis of the knee often experience improvement in knee joint symptoms after endovascular treatment. We considered that it was important to decide the operation indication of lower extremity varices, to know the correlation between the two diseases in the treatment of varicose veins. To investigate the postoperative improvement of knee symptoms related to varicose veins with knee osteoarthritis, we conducted a questionnaire survey for a total of 12 months, from December 2014 to May 2015 and from October 2018 to March 2019. The participants were 35 patients (7 men and 28 women) with varicose veins complicated with knee osteoarthritis. We classified knee osteoarthritis according to a grading scale and compared the improvement of knee symptoms after endovenous thermal ablation. The higher the knee grade, the lower the degree of improvement. However, the improvement was observed in all knee osteoarthritis grades, and as a whole, 25 patients (71.4%) have experienced improvement of subjective symptoms. For patients with knee osteoarthritis, we strongly recommend surgical treatment of the varicose veins regardless of the progression of knee grade. (This is a translation of Jpn J Phlebol 2019; 30(3): 279–283.)

## Introduction

Both osteoarthritis of the knee and varicose veins of the lower limbs are common in women, and their prevalence increases with age. Thus, the patient populations of these conditions overlap, and they often occur together. A common experience in clinical practice is that treating varicose veins in the lower limbs improves knee symptoms by reducing the load on the muscles of the lower leg. However, this is not widely known and few studies have examined it in detail.

In this study, we interviewed patients who complained of knee symptoms at the initial examination to investigate the extent to which subjective knee symptoms improved after endovascular treatment for varicose veins of the lower limbs.

## Participants and Methods

**Figure 1:**
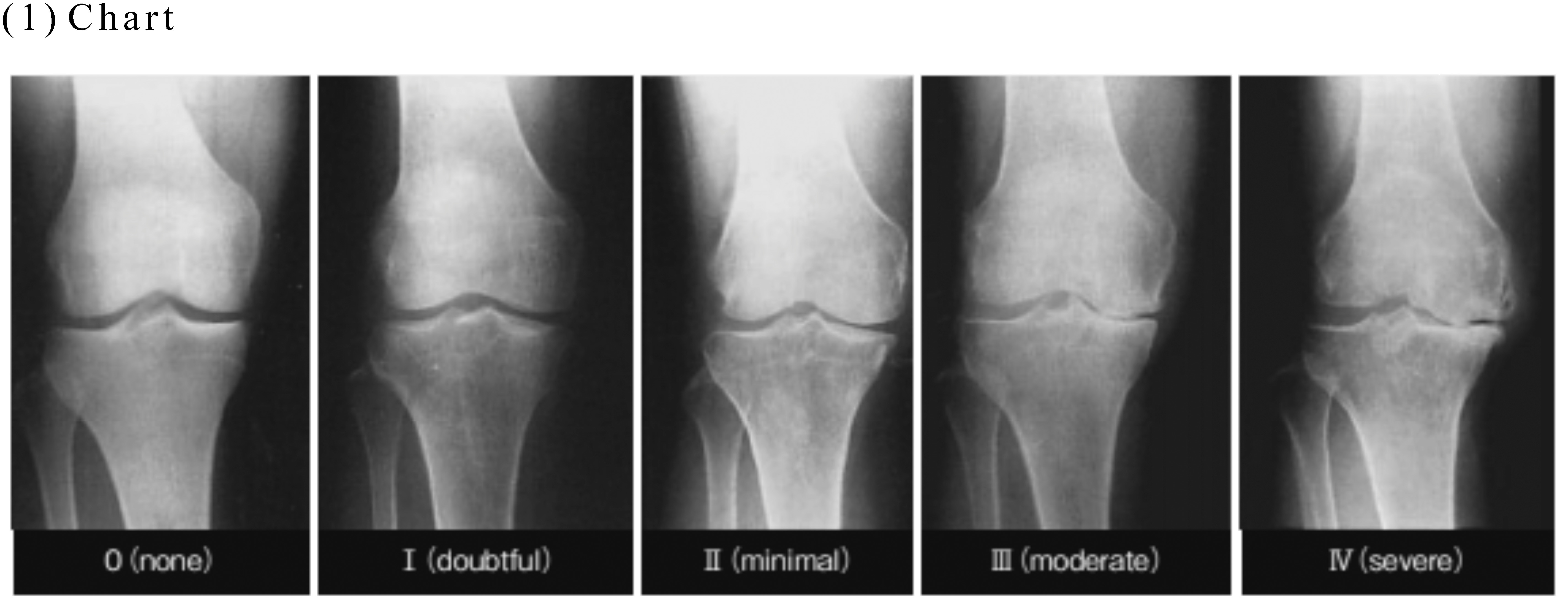
Fig. 1 The Kellgren–Lawrence grade to assess the severity of knee osteoarthritis. Grade 0; no pathological features. Grade 1; doubtful narrowing of the joint space, possible osteophytic lipping. Grade 2; definite osteophytes, possible narrowing of the joint space. Grade 3; moderate multiple osteophytes, definite joint space narrowing, some sclerosis, possible deformity of bone ends. Grade 4; large osteophytes, marked joint space narrowing, severe sclerosis and definite bony end deformity.^3)^

The surveys were conducted for a total of 12 months—6 months from December 2014 to May 2015 and 6 months from October 2018 to March 2019. Ultrasonography of varicose veins of the lower limbs was performed for patients with symptoms of congestion in the lower limbs. Endovascular ablation was performed as recommended in the “Guidelines for Endovascular Treatment for Varicose Veins of the Lower Limbs.”^1)^ The participants were patients who recorded knee pain as a subjective symptom on a questionnaire at the initial visit and who asked to have their knees examined. From these, we selected 35 patients (7 men and 28 women) diagnosed with knee osteoarthritis grades 1–4 of the Kellgren–Lawrence classification^2,3)^ (**Fig. 1**).

They were asked about subjective symptoms of the knee related to the eight items shown in **Table 1**. Changes from the preoperative state at 1 month postoperatively were evaluated using a 4-point scale: improved, slightly improved, unchanged, and worsened.

**Table 1:** Table 1 The questionnaire investigation for knee symptoms that are used in our hospital

Q1	I have a feeling of discomfort in my knee when I start walking in the morning.
Q2	My knee hurts when I put pressure on my knee.
Q3	I cannot sit seiza-style and bend my knee completely.
Q4	My knee hurts when I crouch down.
Q5	My knee is swollen, and I feel the heat sensation.
Q6	I have water on my knee.
Q7	I need to take the knee painkiller.
Q8	I am injected into my joint.

Knee osteoarthritis was diagnosed by radiography. If found, the degree of progression was evaluated using the Kellgren–Lawrence classification system from grade 0 to 4. This system uses standing frontal radiographs, and its main items are narrowing of the joint space and osteophyte formation, as well as the presence or absence of osteosclerosis and deformation. It uses a 5-grade scale from 0 to 4.^4)^ Grade 2 or higher is considered a definitive diagnosis of knee osteoarthritis, but we also included cases of grade 1 (doubtful).

Varicose veins of the lower limbs were evaluated using duplex scanning and air plethysmography. Intravascular ablation was performed on great saphenous veins and small saphenous veins with reflux, along with resection and sclerotherapy as needed.

The degree of improvement was scored as follows. Interview responses of “improved” and “slightly improved” were given 2 points and 1 point, respectively, and “unchanged” and “worsened” were given −1 point and −2 points, respectively. The total score was calculated, and scores of 1 or higher were considered an improvement.

The t-test and chi-square test of independence were used for statistical processing. A p-value of <0.05 was considered a significant difference.

## Results

During the survey period, 460 patients underwent endovascular ablation at our hospital, and 35 patients with knee osteoarthritis (7.6%) were selected as the participants of this study. **Table 2** shows the clinical data on the 35 participants. Similar to the background of knee osteoarthritis patients, the majority were women, who accounted for 80% of the total. Overall, they tended toward obesity with a body mass index of 25.3±4.0 kg/m^2^. The men were more likely to be obese (p<0.05). The grade of the more advanced knee in the Kellgren–Lawrence classification was used for patients who were treated on both legs. There were 10, 13, 5, and 7 cases of grades 1, 2, 3, and 4, respectively.

**Table 2:** Table 2 Demographic data according to the Kellgren–Lawrence grade

	Male (n=7)	Female (n=28)	All (n=35)
Age	69.1±7.7	70.3±6.6	70.0±6.9
Body Mass Index (kg/m^2^)	28.9±4.1	24.5±3.4	25.3±4.0
Kellgren–Lawrence Grade			
Grade 1	0	10	10
Grade 2	4	9	13
Grade 3	2	3	5
Grade 4	1	6	7

The endovascular treatment was radiofrequency ablation in 29 cases and endovenous laser ablation (EVLA) in six cases. The vessels treated were the great saphenous vein in 34 cases and the small saphenous vein in one case.

**Figure 2:**
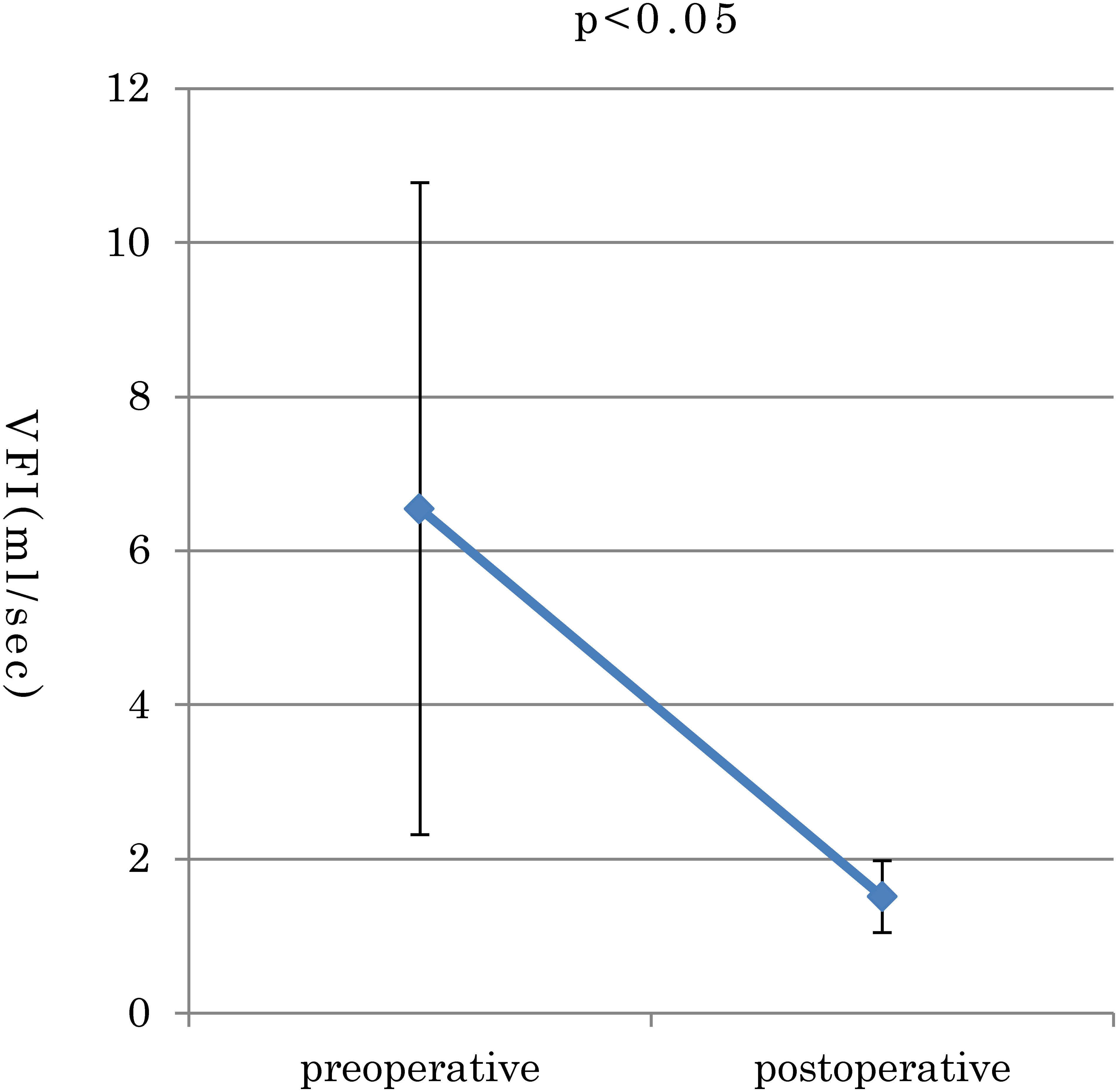
Fig. 2 The average of venous filling index (VFI) at preoperative and postoperative.

Air plethysmography was performed before and after surgery on 27 limbs in 20 patients to calculate the venous filling index (**Fig. 2**). The mean value decreased from 6.55 to 1.51 ml/s (p<0.05), confirming the treatments were effective.

**Table 3** shows the percentage of respondents who said their knee symptoms improved or slightly improved (improvement rates) in each question by knee osteoarthritis grade. There were differences in the improvement rates between the questions (χ^2^: p<0.05). The improvement rate of knee discomfort (question 1) was the highest, with 84% of the total number exhibiting improvement. The improvement rate tended to be high even in more advanced cases. In contrast, the improvement rate for sitting on one’s heels (“seiza” style; question 3) was the lowest, with improvement seen in 36% of the total. The improvement rate tended to decrease as the disease progressed.

**Table 3:** Table 3 Improvement for knee symptoms according to the grade of knee osteoarthritis

	Grade 1 (n=10)	Grade 2 (n=13)	Grade 3 (n=5)	Grade 4 (n=7)	All (n=35)
Q1	6/8	9/10	2/3	4/4	21/25 (84.0%)
Q2	5/8	12/13	2/5	3/5	22/31 (71.0%)
Q3	3/6	4/7	1/5	1/7	9/25 (36.0%)
Q4	6/8	7/9	0/3	4/6	15/24 (65.4%)
Q5	3/5	7/9	1/2	5/6	17/21 (72.7%)
Q6	5/8	5/7	3/4	0/0	13/19 (68.4%)
Q7	1/2	4/6	1/2	1/1	7/110 (63.6%)
Q8	1/3	3/7	1/2	2/5	7/17 (41.2%)

By knee osteoarthritis grade, scores improved postoperatively by +1 point or more in 8 cases of grade 1 (80.0%), 11 cases of grade 2 (84.6%), and 3 cases each for grades 3 (60.0%) and 4 (42.9%). Overall, improvement was observed in 25 patients (71.4%) (**Table 4**). A difference in the degree of improvement was observed between grades 2 and 3. Improvement was observed in 19/23 cases with grades 1 and 2 (82.6%) and in 6/12 cases with grades 3 and 4 (50.0%), which represents significantly more improvement in grades 1 and 2 (p<0.05).

**Table 4:** Table 4 Improvement score according to the grade of knee osteoarthritis

	Grade 1 (n=10)	Grade 2 (n=13)	Grade 3 (n=5)	Grade 4 (n=7)	All (n=35)
Average score	2.9±5.7	4.2±5.0	0.6±2.9	1.9±4.5	2.9±5.0
Improved people	8	11	3	3	25
	80.0%	84.6%	60.0%	42.9%	71.4%
	19		6		*p*<0.05
	82.6%		50.0%		

## Discussion

Patients with varicose veins in the lower limbs are mostly middle-aged and older women, similar to those with knee osteoarthritis. Patients with knee osteoarthritis have significantly higher prevalence of varicose veins in the lower limbs (43% vs. 22%; p=0.015),^5)^ and a relatively large number of patients with varicose veins of the lower limbs are believed to have knee osteoarthritis.^6)^ Obesity is a risk factor for both. As **Table 2** shows, the patients in the present study also tended to be obese. Knee osteoarthritis should be suspected in patients with varicose veins of the lower limbs who are obese and also complain of knee symptoms.

As these conditions are likely to occur together, the following studies have also suggested mutual relationships with regard to therapeutic effects. A study of analgesic effects in 158 knee osteoarthritis patients with and without lower limb venous disease found that damage to peripheral venous blood flow functions reduced the analgesic effects from drugs on knee osteoarthritis.^7)^ Further, in a previous study, we examined subjective symptoms before and after treatment in 431 patients with large varicosities in the great saphenous vein. Improvement was observed in subjective orthopedic symptoms that were not expected to improve preoperatively, including knee and low back pain. Knee pain improved in 18% of the stripping group and 20% of the ELVA group.^8)^

Three typical symptoms of knee osteoarthritis are pain during movement, limited range of motion, and joint swelling, which worsen as the disease progresses. In the early stages, pain is only felt during initial movements, such as when standing up or starting to walk, but when the disease progresses, it becomes difficult to sit on one’s heels and go up and down the stairs.^4)^ On the basis of these symptoms, we created the questionnaire on knee symptoms shown in **Table 1**.

Bone marrow edema is thought to be one cause of knee symptoms in osteoarthritis. Complaints of pain are reported to be more common in patients with bone marrow edema, and pain has been found to become more severe as edema spreads.^9)^ Bone marrow edema is the retention of fluid in extracellular medullary cavities caused by capillary leakage. It is diagnosed with magnetic resonance imaging (MRI). Its main causes are direct invasion of capillaries such as from trauma or tumors, hyperemia, and venous congestion. However, bone marrow edema in knee osteoarthritis is not believed to be caused by one single factor, but rather by a mixture of fibrosis, cell infiltration, micro-fractures, and angiogenesis.^9)^

Treating varicose veins of the lower limbs improves blood congestion in the lower limbs, which reduces lower limb edema and the load on the muscles of the lower leg. In the present study, improvement in knee symptoms after endovascular treatment for varicose veins of the lower limbs is tended to be seen in items on pain during movement and joint swelling, such as knee discomfort and swelling, rather than in items related to limited range of motion, such as difficulty sitting on one’s heels (**Table 3**), and it was suggested that treating varicose veins of the lower limbs also improves the symptoms of blood congestion in the knees. We believe the treatment of varicose veins in the lower limbs alleviates knee pain by improving blood congestion in the knees and, secondarily, by partially reducing bone marrow edema. In the future, we would like to use MRI to quantitatively measure bone marrow edema before and after treatment of varicose veins in the lower limbs.

Looking at the percentages of patients who exhibited improvements by stage and symptom (**Table 4**), we find that the more advanced the knee pathology, the lower the degree of improvement. Compared with Kellgren–Lawrence grade 2, grade 3 exhibits clear narrowing of the joint space, along with some osteosclerosis and possible deformity of bone ends. The degree of improvement in subjective symptoms appears to depend on how advanced these organic changes are. However, it was not unusual for symptoms to improve in grade 4 cases, which indicates that even patients with advanced disease can expect some temporary improvement in their knee symptoms from treating varicose veins of the lower limbs.

In the routine care of varicose veins of the lower limbs, it is important to ask patients about knee symptoms, as this can be an opportunity to diagnose complicating knee osteoarthritis. The fact that treating varicose veins of the lower limbs can improve knee symptoms may provide a rationale for surgery in patients with lower limb varicose veins complicated by osteoarthritis who are unsure whether to undergo surgery. As such, this can be highly significant in the care of both diseases.

## Conclusion

We investigated the therapeutic effects of endovascular treatment for varicose veins of the lower limbs on knee symptoms in patients with complicating knee osteoarthritis. Ablation of varicose veins in the lower limbs improved knee symptoms in patients whose knee osteoarthritis was not advanced.

## Additional Note

This study was approved by the Hiroshima Teishin ethics committee.

## Notes

This is a translation of Jpn J Phlebol 2019; 30(3): 279–283.
